# Lip Filler Versus “Lip Flip”: Longitudinal Public Interest and a Brief Review of Literature

**DOI:** 10.1111/jocd.70048

**Published:** 2025-02-12

**Authors:** Raika Bourmand, Sofia E. Olsson, Shirin Soleimani, Arman Fijany

**Affiliations:** ^1^ Anne Burnett Marion School of Medicine Texas Christian University Fort Worth Texas USA; ^2^ Vanderbilt University Medical Center Nashville Tennessee USA

**Keywords:** botox, botulinum toxin, hyaluronic acid, lip augmentation, lip filler, lip flip, nasolabial injection

## Abstract

**Background:**

The lip augmentation market is projected to reach a value of 11.6 billion USD by 2030. Hyaluronic acid (HA) fillers are the most common type due to biocompatibility and reversibility. However, there has been an increase in alternative procedures, such as the “lip flip,” involving the injection of botulinum neurotoxin A (BoNT‐A) into the superior orbicularis oris muscle.

**Aims:**

The purpose of this study is to review the current literature and evaluate the general public interest in dermal lip fillers compared to supralabial BoNT‐A injections over the past decade.

**Methods:**

The Google Trends database was used to collect relative monthly search volume for the terms “lip filler” and “lip flip” over a decade‐long period (January 1, 2014–January 1, 2024). Google Trends data is automatically normalized with a value of 100 indicating maximal search volume and 1 indicating minimal search volume.

**Results:**

Search volume for lip filler and lip flip increased similarly from 2014 to 2024. Lip filler was consistently more searched and had an average 75% increase in relative search volume per month. Meanwhile, “lip flip” had, on average, a 33% increase in search volume per month.

**Conclusions:**

Although lip filler was more frequently inquired about than “lip flip,” the latter increased in popularity over time, reaffirming its popularity as a potential alternative to lip filler. Superior obicularis oris BoNT‐A injections are rising in popularity and may be a valuable option for patients seeking cosmesis with hesitation toward dermal fillers.

## 1 Introduction

Lip shape and proportions are a prevalent topic and an area of expertise for facial plastic surgeons and cosmetic dermatologists. Lip appearance is also important to patients, with the lip augmentation market being valued at 6.5 billion United States Dollars (USD) in 2022 and a projected 11.6 billion USD by 2030 [1]. Two procedures commonly used for lip augmentation include the colloquially termed “lip filler” and “lip flip.” Both procedures are completed to enhance the lips' appearance but do so through different mechanisms (Table 1).

**TABLE 1:** Summary of differences between minimally invasive lip augmentation techniques. BoNT‐A, Botulinum neurotoxin A; USD, United States Dollar.

	Supralabial BoNT‐A injections “lip flip”	Injectable dermal lip filler
Average price range [2]	100 USD or less	500–1000 USD
Average duration of augmenting effects [3, 4]	8–12 weeks	Up to 12 months
Procedure risks [2, 5]	Bruising, swelling, and tenderness at injection sites Orbicularis oris weakness: trouble pronouncing words, drinking through a straw, spitting, whistling, or eating with a spoon	Bruising, swelling, and tenderness at injection sites Surrounding tissue necrosis Filler migration Temporary or permanent blindness Stroke Death

### 1.1 Lip Filler

**FIGURE 1:**
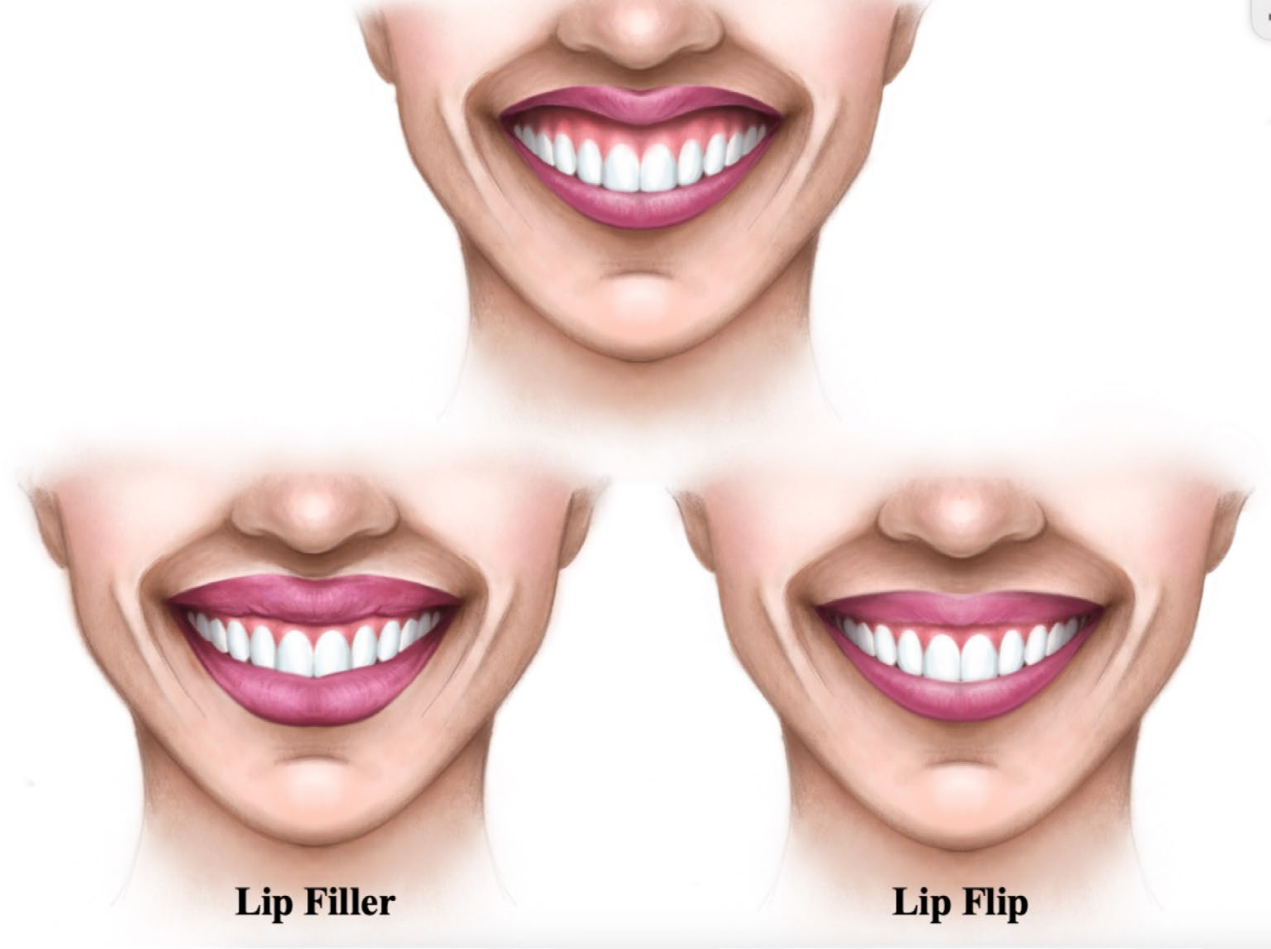
Subtle differences in results from dermal lip fillers and supralabial botulinum toxin injections or “lip flip” for a patient with excessive gingival display and lip retraction.

Approximately 1 million individuals annually choose injectable dermal fillers for facial rejuvenation in the United States (US) [6]. Dermal lip filler treatments involve the administration of an injectable prosthetic directly into the lips to increase their surface area or shape (Figure 1). The most used injectable prosthetic is composed of hyaluronic acid [7]. The history of lip filler dates back to the early 1900s, at which point it was composed of a waxy liquid known as paraffin [8]. Since then, the 1960s introduced silicone as an agent to augment lips, similar to its role in breast augmentation [8]. During the 1980s, there was a transition in the aesthetic medicine community to using collagen as a lip‐filling agent [8]. However, collagen is derived from animals, so allergy testing and additional safety measures are required for individual patients [8]. This initiated a transition to using hyaluronic acid (HA), a soft and gel‐like substance that interacts well with the natural collagen of the lips [8]. Since HA is a naturally occurring substance not derived from animals, it does not require allergy testing and is time‐efficient for patients [8].

Most HA fillers are infused with lidocaine to decrease pain and discomfort during the procedure [6]. The agent also offers the benefit of retaining moisture well within the lips, adding to their overall appearance and health [9]. Patients have control over the volume of HA used and how much they seek to inject, which can be administered gradually until the desired result is achieved. A benefit of HA filler for lip augmentation is its ability to be reversed via dissolution by injectable hyaluronidase [5]. Overall, HA lip filler lasts an average of six to 8 months and is known to cause less bruising or swelling than other dermal fillers. Though relatively safe, adverse effects of HA lip filler can include swelling, contusions, redness, and itching at the injection sites, as well as unsatisfactory aesthetic results [10]. An important adverse effect to this procedure also includes potential movement of substance from the injection site, known as filler migration, which can result in undesirable aesthetic results such as masculinization of the face or facial sagging [5]. The most severe risk associated with using dermal fillers is the injection into a blood vessel, leading to occlusion and poor perfusion to surrounding tissues. This may cause tissue necrosis, blindness, stroke, and rarely death [5].

The Food and Drug Administration (FDA) currently regulates dermal fillers as medical devices [11]. Ideal candidates for lip fillers are individuals who are physically healthy, not pregnant or breastfeeding and are compliant with aftercare procedures such as massaging the lips to prevent clumping [5]. The FDA has only approved dermal fillers for use in adults 22 years of age or older to augment the lips, cheeks, chin, and back of the hand, reducing wrinkles, skin folds, and acne scars and for restoration for facial fat loss for patients with human immunodeficiency virus [5]. The average cost of HA dermal fillers is 715 USD, while the average cost of lip augmentation with dermal fillers is 743 USD [12].

### 1.2 Lip Flip

In contrast, the “lip flip” procedure was first introduced in 2017 and became heavily popularized on the social media platform TikTok, where the #lipflip hashtag gained nearly 64.4 million views by July 2021 [13]. This procedure involves the injection of botulinum toxin A (BoNT‐A) into the oral commissures and superior vermillion border to reduce function of the superior orbicularis oris muscle. BoNT‐A is a neuromodulating agent that prevents muscle excitation by inhibiting the soluble NSF‐attachment protein receptor (SNARE) proteins necessary for acetylcholine release into the neuromuscular junction [3]. While BoNT‐A injections are approved for cosmetic indications such as reducing forehead wrinkles, frown lines, and crow's feet, they are also used for “off‐label” cosmetics such as lip flips [3]. This involves injection of BoNT‐A immediately superior to the upper lip at the center and corners of the mouth. Relaxation of local muscular anatomy, such as the orbicularis oris muscle, provides a fuller gross appearance of the lips [14]. Hence, the procedure does not offer increased fullness and surface area of the lips but rather the formation of a “pout” in the facial expression [3].

Supralabial BoNT‐A injections can also be used with dermal fillers to accentuate results. This procedure differs from dermal lip fillers, where injections containing a filling agent are directly administered into the lips. Therefore, supralabial BoNT‐A injections in the “lip flip” are intended to produce a more natural and subtle result than HA fillers [14]. Hence, the benefits of the “lip flip” include subtly defining one's upper lip, preventing and reducing vertical lines that form above the upper lip, and decreasing the amount of superior gingiva shown while the patient smiles (Figure 1) [3]. The “lip flip” lasts an average of 8–12 weeks due to the temporary effects of BoNT‐A, which, in comparison to the lifespan of lip filler, lasts up to 12 months [3]. Similar to lip filler, adverse side effects of the “lip flip” procedure include swelling and bruising at the injection site [15]. Since BoNT‐A is injected supralabially to create the illusion of fuller lips, it does not directly address the lower lip.

“Lip flips” are ideal for patients who desire fuller lips but do not want lip fillers, have excessive gingival display while smiling, have an upper lip that retracts with their smile, or prefer relatively short‐term or temporary results [3]. Additionally, unlike lip filler, the “lip flip” does not require post‐procedural aftercare or compliance, allowing patients to return to normal activities immediately [2]. It is essential to consider that BoNT‐A is a neurotoxin and hence presents with risks associated with neuromuscular inhibition, such as trouble pronouncing certain words, drinking through a straw, spitting, whistling, or eating with a spoon. Therefore, this procedure may not be ideal for those who professionally sing or speak and risk further impacting their daily activities from temporarily impaired lip function [2].

An important consideration for patients when comparing supralabial BoNT‐A injections to dermal lip filler is the cost. The “lip flip” typically requires between 4 and 6 units of BoNT‐A, costing 10–15 USD each. Thus, a “lip flip” normally costs up to 100 USD, in comparison to lip filler costing between 500 and 1000 USD per full syringe (Table 1) [2].

The difference between these procedures and the same goal of lip augmentation is that they allow for diverse options that best fit individualized patient goals.

It is critical to have a comprehensive understanding of public interest in procedures for all patients, clinicians, and stakeholders involved. Recently, researchers have been using the Google Trends database to assess public interest through search queries [16]. Google has been acknowledged as the most widely used search engine globally, but more specifically, in the United States, which occupies nearly 87% of the search engine market [17]. Therefore, search trends, as reported by Google, are more representative of the general population than less used alternative search engines. This study is the first to demonstrate a longitudinal analysis evaluating the shift in public interest in minimally invasive lip augmentation techniques described by the Google Trends database.

## 2 Materials and Methods

The two search terms “Lip Filler” and “Lip Flip” were identified with a consensus among the authors due to their everyday use among the general population. Since 2000, surgical lip augmentation has been reported to have increased by 60%, while lip filler has explicitly increased by 312% [18]. With the increased interest in dermal fillers as a minimally invasive approach, the term “Lip Flip” was chosen compared to an alternative minimally invasive technique for lip augmentation. Ultimately, this study aims to assess and compare the growing interest in minimally invasive lip augmentation techniques as described by Google Trends.

Monthly search volumes for the terms over a decade were evaluated using historical data from the Google Trends database. Google Trends provides information on relative search volume at any given time and has been utilized in recent scientific literature to investigate public interest without barriers to purchase. This study gathered search volume data from January 1, 2014 to January 1, 2024 in the United States. Google Trends presented the relative monthly search volumes of these terms on Google as normalized values ranging from 0 to 100. A 0 value represents minimal search volume, with 100 representing the highest. Relative values were plotted over time using the Microsoft Excel software and descriptive analysis was performed. Paired *t*‐testing was performed on the relative search volumes over time for each term to determine significance as defined by a *p*‐value ≤ 0.05.

## 3 Results

**FIGURE 2:**
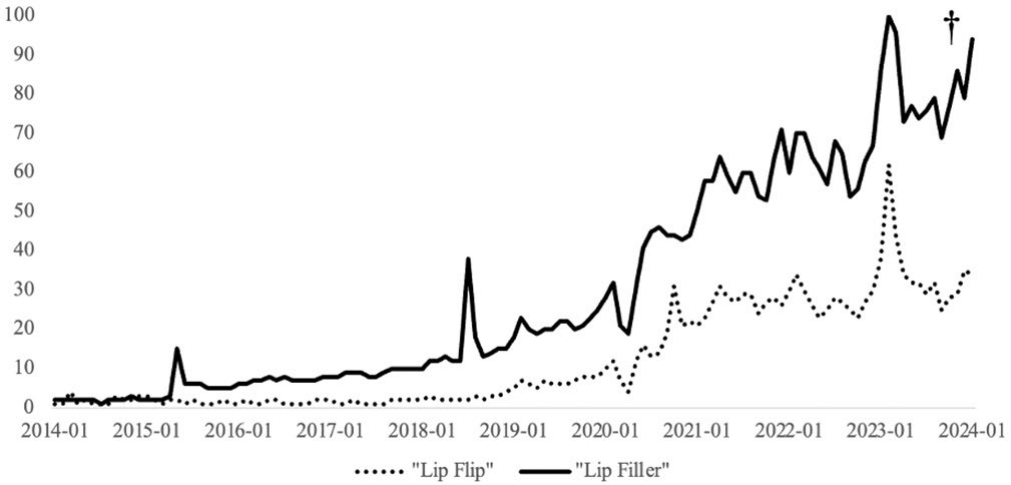
Relative monthly search volume of “Lip Filler” and “Lip Flip” on Google from January 1, 2014 to January 1, 2024 (^†^ *p* < 0.001).

In the past decade, search volume for both “Lip Filler” and “Lip Flip” has increased similarly (Figure 2). “Lip Filler” was consistently more searched and had an average 75% increase in relative search volume per month. Meanwhile, on average, “Lip Flip” had a 33% increase in search volume per month. The average relative search volume for “Lip Flip” was quantified as 12.1 while “Lip Filler” was 30.4. Patterns in search volume were also relatively consistent between the two search terms. For example, both terms experienced a rapid decline between February 2020 and April 2020 (67% “Lip Flip” and 41% “Lip Filler”). Both terms also underwent a rapid increase in search volume between September 2022 and February 2023 (148% “Lip Flip” and 85% “Lip Filler”). However, “Lip Flip” consistently had a lower relative Google search volume over time (*p* < 0.001).

## 4 Discussion

This study is the first to analyze the longitudinal public interest in dermal lip fillers and supralabial BoNT‐A injections as described by Google Trends data. In the past decade, search volume for both “Lip Filler” and “Lip Flip” demonstrated similar upward trends. However, “Lip Filler” consistently dominated in search volume, showing an average increase of 75% in relative search volume per month compared to a 33% monthly increase for “Lip Flip.” The average relative search volume over the study period was quantified at 30.4 for “Lip Filler” and 12.1 for “Lip Flip,” highlighting the greater popularity of “Lip Filler.” Temporal patterns in search volume were also consistent between the two terms. For instance, both experienced a sharp decline between February 2020 and April 2020, with “Lip Flip” searches decreasing by 67% and “Lip Filler” by 41%. Conversely, both terms saw a marked increase in search interest between September 2022 and February 2023, with relative search volumes rising by 148% for “Lip Flip” and 85% for “Lip Filler.” Despite these parallel trends, “Lip Flip” maintained a consistently lower relative Google search volume throughout the observed period, with this difference reaching statistical significance (*p* < 0.001). These findings suggest that while interest in “Lip Flip” is growing, in terms of public interest, it remains secondary to “Lip Filler”. We would like to emphasize the importance of holistic ethical considerations in the promotion of cosmetic procedures, such as medical needs, patient desires, and safety rather than solely considering public interest or popularity.

Dermal lip fillers may have demonstrated greater increases in public interest for several reasons, including their long‐standing presence as a minimally invasive technique for lip augmentation, their ability to correct asymmetries of the lips to enhance proportionality, and increased familiarity among patients with this procedure. Additionally, lip filler is an effective procedure in reducing visible aging and the appearance of wrinkles or fine lines. This may be particularly interesting among those who smoke or develop vertical wrinkles that travel upward from the superior lip border [19]. Moreover, this procedure can define lip shape or create a cupid's bow outside of adding natural fullness to the lips. A unique advantage of dermal lip fillers, which may provide substance for its popularity over the “lip flip,” is the ability to reverse the procedure through injection of hyaluronidase, an enzyme with the ability to dissolve intradermal HA [11].

However, dermal lip fillers can also pose reasons for which patients may choose not to undergo this procedure. For example, a recent study assessing the three popular types of lip filler found that the formation of nodules was a common complication across all kinds [20]. Other complications reported in the study included HA migration to other face areas, skin discoloration, and herpetic outbreaks, which, while generally rare, can result in considerable patient dissatisfaction. While hyaluronidase can reverse lip filler, it does carry risk of allergic reactions or local inflammation which must also be considered in clinical settings [20]. In comparison, the “lip flip” offers similar benefits to lip filler as it can be used to confront fine lines of aging and benefit the aesthetics of the lips. However, the lower public interest may be partly explained by the irreversible nature of this procedure immediately after it is performed. Once injected, patients must wait 3–4 months for the BoNT‐A to wear off naturally [2]. Although it is infrequently associated with adverse effects such as perioral muscular palsy and muscular incompetence, this can significantly impact one's quality of life by presenting issues of drooling or difficulty speaking. The results of the “lip flip” are also less customizable than dermal filler which may limit appeal of precision in enhancement [21]. Overall, both procedures are generally regarded as safe when performed by skilled practitioners; however, careful patient selection and thorough consultation are important in mitigating risk and ensuring patient satisfaction.

Socioeconomic factors are a critical part of aesthetic medicine as well. A recent study, which assessed 915 insurance plans across the United States, reported that none covered neurotoxins like BoNT‐A [22]. Only 72 potentially covered collagen injections under the requirement of demonstrating medical necessity or significant variation of physical appearance from the patient's experienced gender for gender affirmation [22]. Of these 72 plans under the Affordable Care Act, 69 outlined exclusions due to cosmetics [22]. Based on the idea that lip injections are primarily an out‐of‐pocket expense, it is vital to assess how patients decide to pursue these procedures. Nationwide, it has been found that there has been a historical association between the demand for cosmetic procedures and the performance of the US economy [23]. From 2006 to 2020, the American Society of Plastic Surgeons found that gross domestic product (GDP) per capita year‐over‐year change was positively correlated with case volume [23]. In the US, the mean age of those undergoing cosmetic procedures was found to be 45–64 years old, with women undergoing more cosmetic surgery than men. The overall percentage in procedure frequency showed a nearly 2% decline among White patients and an increase among Black, Hispanic, Asian, and Native American patients by 7.5%, 4.7%, 14.5%, and 105.5%, respectively [24]. In addition to these factors, economic status has also demonstrated a strong relationship with the prevalence of cosmetic procedures. In the US, the median household income of people who had undergone cosmetic procedures was most commonly in the highest quartile [24]. Importantly, the reasons to pursue minimally invasive lip augmentation are complex and multifaceted with cultural beauty standards, social trends, personal preferences, economic contributors, and marketing influences all playing impactful roles. We encourage future research to explore these factors and their influence on minimally invasive lip augmentation procedures.

A strength of this study is its broad inclusivity—with Google being the US' most widely used search engine—accounting for nearly 87% of internet searches [18]. Additionally, colloquial search terms allowed for better representation of the general public, given that the use and understanding of medical jargon may be limited outside of the medical community [25]. A limiting factor to this methodology is that it does not provide insight on an individual basis surrounding why such terms were searched. Users who use a search engine other than Google are not represented in the study and may display alternative search trends or habits. Additionally, the use of the Google Trends database as a sole data source limits information on patient demographic factors, satisfaction, and outcomes. Therefore, the present study does not account for the potential biases inherent to search engine data, such as geographic and socioeconomic variations in search habits. The inclusion of alternative methodologies such as patient surveys or clinical data could enhance the validity of the findings, as well as provide a more comprehensive understanding of the trend data. Addressing these limitations would provide a more nuanced prospective on the public's evolving preferences for these procedures. This reveals a need for future research surrounding patient and physician education on minimally invasive lip augmentation procedures and what deciding factors are considered, such as safety, cost, or convenience. It also emphasizes the need for future research assessing sociodemographic factors that play a role in cosmetic lip augmentation, ultimately allowing us to understand better the populations seeking these procedures and their accessibility. Future directions should include the integration of both patient and practitioner perspectives which could provide critical insight into decision‐making processes and preferences. The incorporation of clinical data on outcomes and trends could complement such perspectives, to offer a more comprehensive understanding of the topic and relevance of the research findings.

## 5 Conclusion

Overall, this Google Trends study comparing public interest in the terms “lip filler” versus “lip flip” over a decade reveals a greater increase in interest in dermal lip fillers. This trend emphasizes the popularity and acceptance of minimally invasive lip augmentation procedures in aesthetic medicine. The consistent increase in search queries for both terms indicates a general societal interest in enhancing lip volume and shape, potentially driven by cultural norms and advancements within cosmetic medicine. While “lip flip” showed a slower increase, it suggests a niche appeal to those interested in more subtle augmentation. This study informs future research, which can advance the techniques used for lip augmentation to consider patient cosmetic desires with maximum patient safety and satisfaction.

## Author Contributions

R.B. contributed to data collection, writing of the original manuscript, review, and editing. S.E.O. contributed to data analysis, writing of the original manuscript, review, and editing. S.S. contributed to figure design and manuscript review and editing. A.F. contributed to manuscript review and editing.

## Ethics Statement

The authors have nothing to report.

## Conflicts of Interest

The authors declare no conflicts of interest.

## Data Availability

The data that support the findings of this study are available in Google Trends at https://trends.google.com/trends/. These data were derived from the following resources available in the public domain:—Google Trends, https://trends.google.com/trends/explore?date=2014‐01‐01%202024‐01‐01&geo=US&q=lip%20flip,lip%20filler&hl=en.
